# Selection of fusion levels using the fulcrum bending radiograph for the management of adolescent idiopathic scoliosis patients with alternate level pedicle screw strategy: clinical decision-making and outcomes

**DOI:** 10.1371/journal.pone.0120302

**Published:** 2015-08-13

**Authors:** Dino Samartzis, Yee Leung, Hideki Shigematsu, Deepa Natarajan, Oliver Stokes, Kin-Cheung Mak, Guanfeng Yao, Keith D. K. Luk, Kenneth M. C. Cheung

**Affiliations:** Department of Orthopaedics and Traumatology, The University of Hong Kong, Pokfulam, Hong Kong, SAR, China; University of Michigan, UNITED STATES

## Abstract

**Objective:**

Selecting fusion levels based on the Luk *et al* criteria for operative management of thoracic adolescent idiopathic scoliosis (AIS) with hook and hybrid systems yields acceptable curve correction and balance parameters; however, it is unknown whether utilizing a purely pedicle screw strategy is effective. Utilizing the fulcrum bending radiographic (FBR) to assess curve flexibility to select fusion levels, the following study assessed the efficacy of pedicle screw fixation with alternate level screw strategy (ALSS) for thoracic AIS.

**Methods:**

A retrospective study with prospective radiographic data collection/analyses (preoperative, postoperative 1-week and minimum 2-year follow-up) of 28 operative thoracic AIS patients undergoing ALSS was performed. Standing coronal/sagittal and FBR Cobb angles, FBR flexibility, fulcrum bending correction index (FBCI), trunkal shift, radiographic shoulder height (RSH), and list were assessed on x-rays. Fusion level selection was based on the Luk *et al* criteria and compared to conventional techniques.

**Results:**

In the primary curve, the mean preoperative and postoperative 1 week and last follow-up standing coronal Cobb angles were 59.9, 17.2 and 20.0 degrees, respectively. Eighteen patients (64.3%) had distal levels saved (mean: 1.6 levels) in comparison to conventional techniques. Mean immediate and last follow-up FBCIs were 122.6% and 115.0%, respectively. Sagittal alignment did not statistically differ between any assessment intervals (p>0.05). A decrease in trunkal shift was noted from preoperative to last follow-up (p = 0.003). No statistically significant difference from preoperative to last follow-up was noted in RSH and list (p>0.05). No "add-on" of other vertebra or decompensation was noted and all patients achieved fusion.

**Conclusions:**

This is the first report to note that using the FBR for decision-making in selecting fusion levels in thoracic AIS patients undergoing management with pedicle screw constructs (e.g. ALSS) is a cost-effective strategy that can achieve clinically-relevant deformity correction that is maintained and without compromising fusion levels.

## Introduction

The main goals of surgical treatment of thoracic adolescent idiopathic scoliosis (AIS) is to achieve curve correction, obtain a balanced spine, and to improve cosmesis.[1–6] As such, instrumented fusion of the spine over multiple levels for AIS is performed [7]. The use of pedicle screws for AIS treatment are commonly utilized because they are a powerful tool to provide stability and three-dimensional deformity correction.[7–12]

In order to achieve the aims of AIS surgical treatment, selection of fusion levels is important. Although throughout the years, there have been several reports as to the ideal strategy of selection of fusion levels,[13–20] the method as proposed by Harrington in the 1970s continues to be widely used.[21] According to Harrington, the way to ensure a parallel fusion block, and prevent the adding-on phenomena and decompensation was to fuse from a horizontal vertebra above to a horizontal vertebra below the curve.[21, 22] However, this method entails relatively long fusion blocks that can also present complications in the long-term. For example, the long fusion block may increase stress at the adjacent non-fused level, which may lead to degenerative changes (e.g. narrowing of the lumbar disc spaces, sclerosis of the endplate and facet joints, osteophyte formation) in spinal segments beneath fusion may occur and subsequent back pain. Also, this methodology was not based on "curve flexibility" or the use of pedicle screw fixation strategies. Studies have shown that curve flexibility and the use of pedicle screws have a predictive role in determining the degree of postoperative curve correction; thereby, contributing to selective thoracic fusion in AIS patients.[23]

Selective thoracic fusion utilizing pedicle screws may preserve motion segments as well as decrease costs and neurological risk associated with the use of additional pedicle screws while maintaining the goals for the surgical treatment of AIS. [7, 24] As such, clear decision-making in determining the fused levels for selective thoracic fusion is needed. Currently, several techniques have been reported that help assess curve flexibility and thereby facilitate level selection for fusion, such as supine bending,[25] push prone, [26, 27] and traction radiography. [28, 29] However, although these are promising techniques, they have inherent limitations. For example, with respect to push prone and traction radiography, a physician is needed to apply the pressure or the traction force. The force exerted is difficult to standardize for every patient, the health-care worker is exposed to ionizing radiation, and at times it presents a challenge for advanced preoperative planning and consultation with the patient. With regards to the supine bending radiograph, the degree of curve flexibility is patient-dependent.

**Fig 1:**
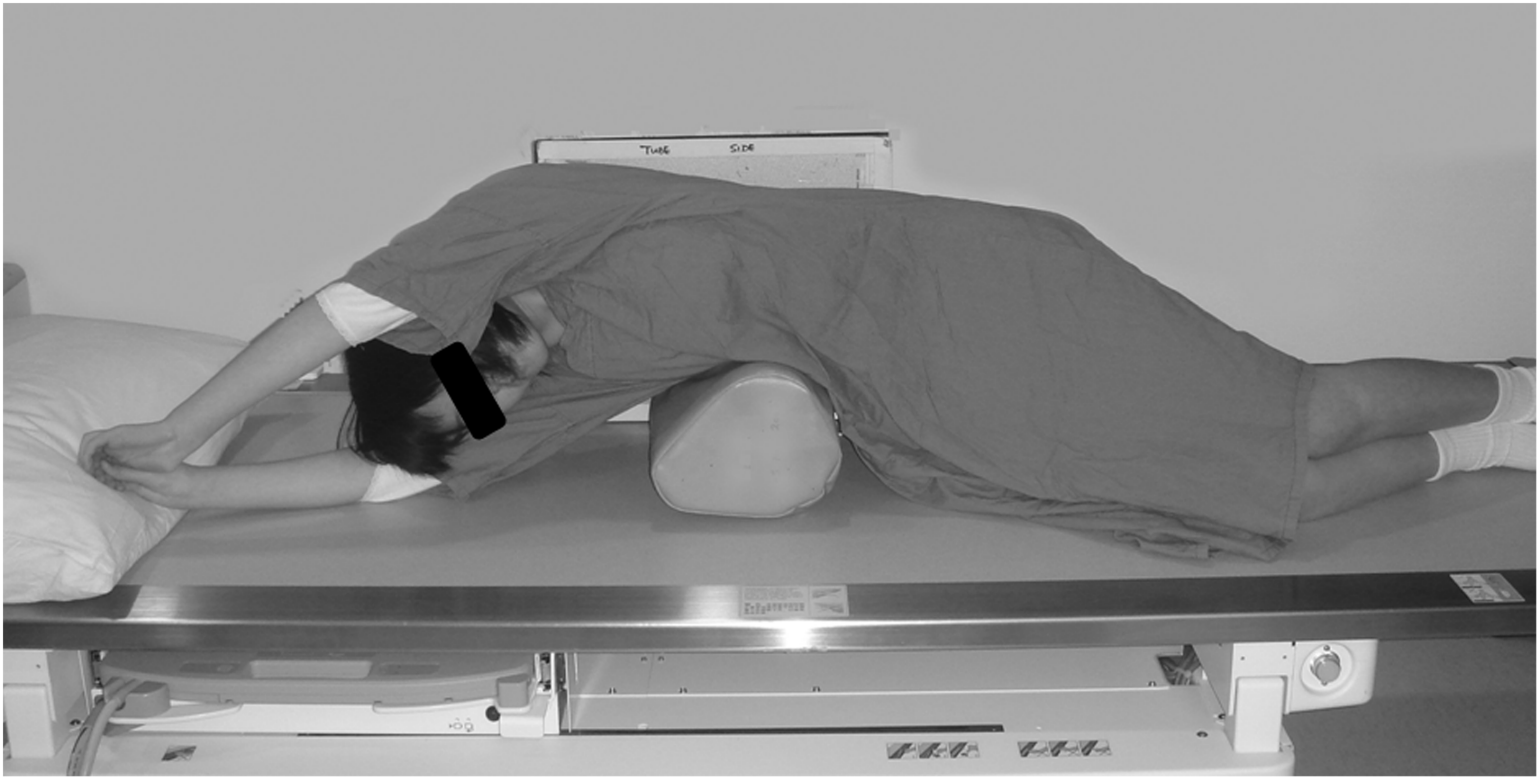
Fulcrum bending radiograph. The patient is positioned on the lateral decubitus position. A padded cylinder (fulcrum) of appropriate size is placed on the side of the curve at the level of the rib corresponding to the apex of the curve. For example, if the apex vertebra of the curve is at T9, the fulcrum should be placed at the T9 rib. The fulcrum should be positioned to allow the shoulder and the pelvis to be lifted off the table.

To counter the limitations associated with various radiographic techniques to assess curve flexibility, the fulcrum-bending radiograph (FBR) (Fig 1) was developed by the authors and has shown to maintain a strong predictive utility in determining postoperative curve correction and facilitating selection of fusion levels in hook and hybrid systems.[23, 30–36] Furthermore, based on the FBR, the fulcrum bending correction index (FBCI) can also be determined, which is a function of the correction rate taking into account the curve's flexibility.[32] Therefore, based on the FBR, the authors have previously shown that the more flexible the curve, the desired postoperative correction is more easily attainable and predictive.[32] With this in mind and with the advent of pedicle screw fixation, curve flexibility may play a role in the clinical decision-making for shorter fusion. This concept was further supported by Luk *et al* [33] noting that 60% of levels were saved when selection of fusion levels was based on the FBR in hook and hybrid systems (i.e. pedicle screws, sublaminar wire, and hooks) as compared to the Harrington method. In addition, based on their two year follow-up, utilizing this method, Luk *et al* [33] did not note any add-on phenomena or decompensation.

A number of studies have shown significant differences between multiple pedicle screw anchor points and hybrid systems for the surgical treatment of thoracic AIS.[7, 8, 37] However, the rationale for using multiple and contiguous level pedicle screws remains unclear, in particular for more flexible curves.[31] In addition, with the additional use of each pedicle screw, this increases the risk of intraoperative complications and implant costs. In order to maintain a balance between good correction, costs and complication risk, we have reported on the short-term safety and efficacy of utilizing alternate level screw strategy (ALSS) pedicle fixation for thoracic AIS in the setting of the FBR. [23]

The FBR has been shown to accurately determine fusion levels using the hook and hybrid systems.[33] However, it remains unknown whether utilizing the authors’ technique of selecting fusion levels based on the FBR preserves motion segments in comparison to traditional fusion level selection strategy (i.e. Harrington method) while achieving and maintaining curve correction in AIS patients with ALSS. Therefore, the aim of this study was to prospectively verify whether a pedicle screw system, ALSS, adequately achieves and maintains correction if the selected fusion level was based on the FBR in comparison to the conventional Harrington method.

## Materials and Methods

The study was conducted at a children's hospital, largely managed by the authors’ Department of Orthopaedics and Traumatology. At the time of the study, Institutional Review Board (IRB) approval was not required. Due to the retrospective nature of the study as to when the imaging and clinical information was obtained and the subsequent prospective re-visitation of the imaging for data extraction, IRB approval was not required by our institution. However, patients and parents understood that the hospital was a teaching hospital affiliated with a university and that research is continuously being conducted. All the data was analysed anonymously with respect to patient identity. Data collection and extraction was performed by individuals not involved with the clinical care of the patients.

A retrospective study with prospective radiographic data collection and analyses was performed. We assessed consecutive patients with main thoracic AIS who underwent single stage posterior only correction and instrumentation with ALSS from 2005 to 2008 at the Duchess of Kent Children's Hospital in Hong Kong. Each patient underwent pedicle screw fixation via an ALSS with titanium rods. In line with the Cheung *et al* [23] technique of ALSS, in all cases, pedicle screws were inserted bilaterally at alternate levels with fixation points at both end vertebrae in the fusion block. In the event of even number of levels in the fusion block, screws were also placed at the adjacent vertebra to the distal end vertebra. Patients with ALSS who obtained up to two year postoperative follow-up were included in the study.

Assessment of the curve's flexibility was based on the FBR (Fig 1), whose technique has been previously described.[23, 30–36, 38] In general, each patient was positioned on the lateral decubitus position. A padded cylinder (fulcrum) of appropriate size was placed on the side of the curve at the level of the rib corresponding to the apex of the curve. For example, if the apex vertebra of the curve was at T9, the fulcrum was placed at the T9 rib. The fulcrum was positioned to allow the shoulder and the pelvis to be lifted off the table.

Selection of fusion levels was based on the protocol reported by Luk *et al*.[33] In general, based on anteroposterior plain radiographs, a parallel line was drawn at the inferior endplate of the estimated distal instrumented vertebra (DIV). From the line above the center of the DIV, a perpendicular line was erected, referred to as the center line (CL). After the estimated proximal instrumented vertebra (PIV) was identified, a parallel line was drawn at the superior endplate. The Cobb angle is determined based on DIV and PIV. If the shift from the PIV was greater than 20 mm from the CL, the next caudal vertebra was chosen as the DIV and these were the selected levels for instrumentation. However, if the shift was less than 20 mm and the Cobb angle was greater than 20 degrees, then the next cranial vertebra was chosen as the estimated PIV.

We assessed pre- and postoperative immediate (1 week) and last follow-up (minimum 2 years) Cobb angles of standing coronal and preoperative FBR. Other parameters measured were the sagittal profile (T5-T12) in degrees as well as the trunkal shift, list and radiographic shoulder height (RSH) in millimetres (mm). Trunkal shift was defined by measuring the perpendicular distance from the centre sacral line to a line that bisects the distance from the lateral edges of the rib margins in the mid thoracic level.^10^ List was defined as the degree of deviation from the S1 spinous process from a vertical line drawn from the C7 spinous process. RSH was defined as the side-to-side difference in height of the intersection points between the upper surface of the clavicle and a vertical line drawn at 10cm from the midline division of the medial ends of the clavicle, derived from the standing postero-anterior radiograph.^10^ With the exception of the FBR performed preoperatively, all radiographic parameters were assessed preoperative and postoperatively at 1 week and 2 years. All radiographic measurements were ascertained using a DICOM based Radworks 5.1 (Applicare Medical Imaging BV, Zeist, The Netherlands) computer software program. Individuals trained in radiographic assessment of the above parameters obtained the imaging data (DS, YL, DN, HS, OS). Any data collection discrepancies were discussed and a consensus reached.

Based on the radiographic assessments, the correction rate, fulcrum flexibility and FBCI were obtained. The following equations illustrate how these values were derived:

$$Correction\;Rate\;\%\;=\frac{\left(\text{Preoperative Cobb Angle }-\text{ Postoperative Cobb Angle}\right)}{\text{Preoperative Cobb Angle}}\times \;100$$

$$Fulcrum\;Flexibility\;\%=\frac{\left(\text{Preoperative Cobb Angle }-\text{ Fulcrum Bending Cobb Angle}\right)}{\text{Preoperative Cobb Angle}}\times \;100$$

$$Fulcrum\;Bending\;Correction\;Index\;\%\;=\frac{\left(\text{Correction Rate}\right)}{\text{Fulcrum Flexibility}}\times \;100$$

With regard to the number of distal levels saved for the fusion block, comparison was made with the conventional Harrington method, which aims to obtain a parallel fusion block and prevent decompensation by fusing from a horizontal vertebra above to a horizontal vertebra below the curve as previously described.[21, 22] Any evidence of instrumentation-related complications, presence of radiographic fusion, and add-on of other vertebra was also noted. In addition, the Lenke *et al* [19]classification scheme was used to categorize the curve-type of the spinal deformity for each patient.

### Statistical Analysis

SPSS v14 (Chicago, Illinois, USA) statistical software was utilized to acquire descriptive and frequency statistics of the data. Non-parametric comparison statistics on the measurement parameters (coronal and sagittal alignments, FBCI, trunkal shift, list, and RSH) were performed comparing the amount of correction achieved at various intervals of postoperative follow-up with those measured at the preoperative period. The number of distal levels saved was calculated by comparing the final levels with that achieved using conventional techniques (i.e. Harrington method). On the basis on the number of patients in our study, their variations based on preoperative and last follow-up coronal alignments, and our level of significance, a 100% statistical power was present to eliminate the chance of a Type II error. The threshold for statistical significance was established at p<0.05.

## Results

Twenty-eight patients (n = 8 males; n = 20 females) were eligible for inclusion into the study, of which all were included in the study and further assessed. The mean age at surgery was 14.5 years (range: 11–21). Based on the radiographic classification of deformity curve type by Lenke *et al*,[19] patients in this study had Lenke type 1A (n = 8, 28.6%), type 1B (n = 13, 46.4%) or type 1C (n = 7, 25.0%) curves.

The radiographic findings are noted in Tables 1 and 2. In the primary curve, the mean preoperative, immediate postoperative and last follow-up standing coronal Cobb angles were 59.9, 17.2, and 21.6 degrees, respectively. There was a statistical significant difference from preoperative to immediate postoperative coronal Cobb angle, (p<0.001) as was there from immediate to postoperative last follow-up (p<0.001). The mean preoperative FBR was 23.9 degrees.

**Table 1:** Demographic and radiological data obtained at pre operative and post operative time periods (immediate and minimum 2 year follow-up).

Parameters	Mean (±SD; Range)
**Baseline age (years)**	**14.5** (2.5; 11–21)
**Preoperative**	
** Standing PA Cobb Angle (degrees)**	**59.9** (10.3; 49.1–92.6)
** FBR Cobb Angle (degrees)**	**23.9** (10.2; 6.9–47.6)
** FBR Flexibility (%)**	**60.5** (17.5; 26.8–86.5)
** Sagittal Alignment Cobb Angle (degrees)**	**18.1** (10.0; 3.8–46.0)
** Number of Levels Fused**	**8.4** (1.2; 7.0–11.0)
**Postoperative (Immediate)**	
** Standing PA Cobb Angle (degrees)**	**17.2** (7.6; 3.2–30.0)
** Correction Rate (%)**	**71.1** (12.8; 47.5–93.5)
** FBCI (%)**	**122.6** (30.1; 78.2–216.4)
** Sagittal Alignment Cobb Angle (degrees)**	**16.1** (7.3; 4.0–37.1)
**Postoperative (Last Follow-up)**	
** Standing PA Cobb Angle (degrees)**	**20.0** (7.2; 7.5–32.0)
** Correction Rate (%)**	**66.1** (12.8; 37.0–90.8)
** FBCI (%)**	**115.0** (35.3; 63.7–239.2)
** Sagittal Alignment Cobb Angle (degrees)**	**17.2** (6.0; 5.2–26.9)

**Table 2:** Summary of the physical parameters at pre-operative and post-operative follow up periods (immediate and minimum 2 year follow-up). All measurements were done in millimeters. RSH = radiographic shoulder height.

Parameter	Mean (±SD; Range)
**Preoperative**	
** Trunk shift**	**23.9** (16.2; 1.0–60.0)
** RSH**	**7.7** (5.3; 2.0–20.0)
** Listing**	**12.6** (10.7; 0–40.0)
**Postoperative (Immediate)**	
** Trunk shift**	**15.3** (11.3; 1.0–59.0)
** RSH**	**17.4** (8.4; 2.0–33.0)
** Listing**	**13.6** (13.5; 1.0–63.0)
**Postoperative (Last follow-up)**	
** Trunk shift**	**10.0** (6.7; 1.0–40.0)
** RSH**	**11.4** (8.4; 1.0–40.0)
** Listing**	**15.0** (12.1; 1.0–44.0)

**Fig 2:**
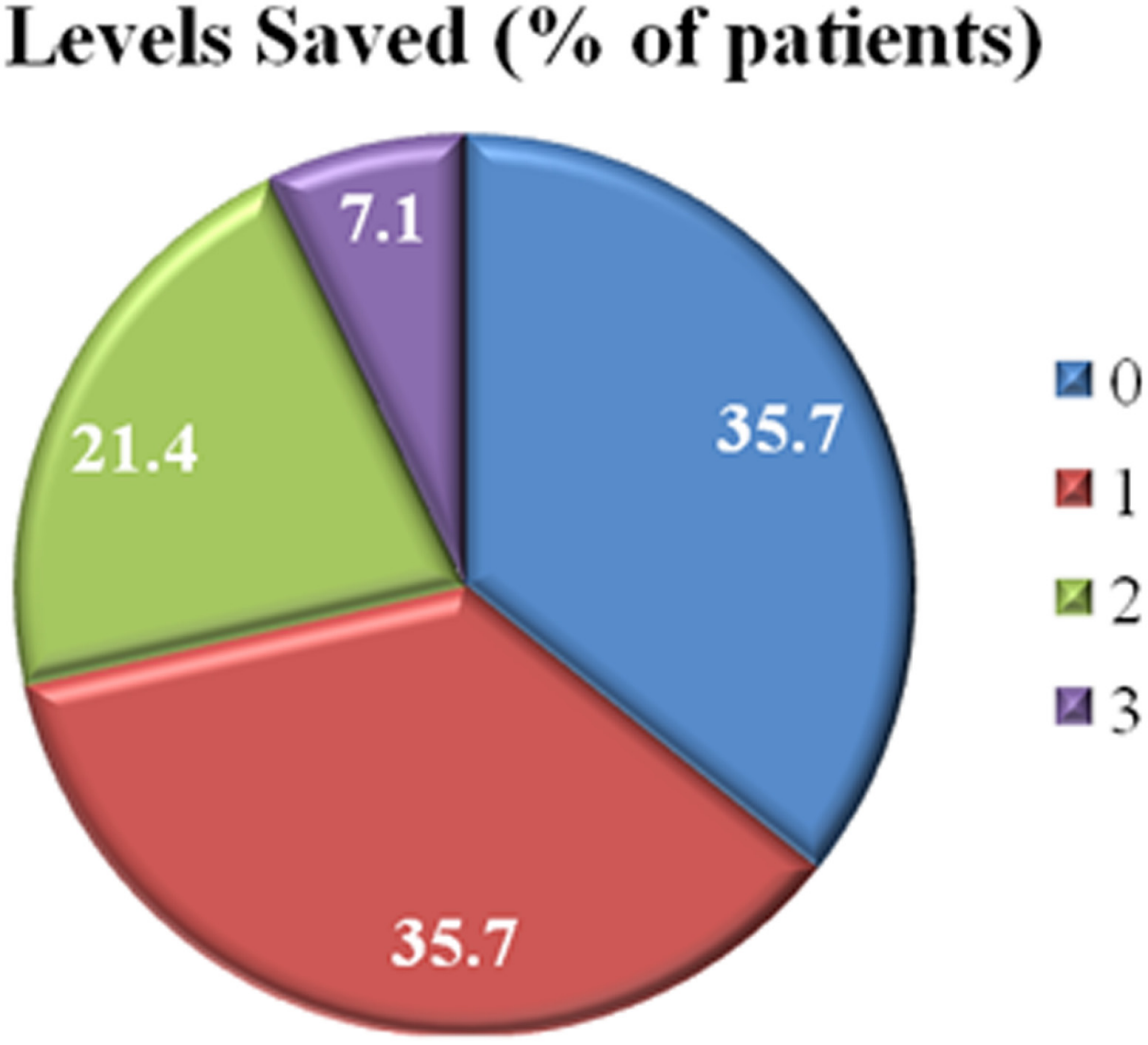
Pie chart illustrating the number of levels saved of all patients using the fulcrum bending radiograph to select the fusion levels when alternate level pedicle screws are inserted.

**Fig 3:**
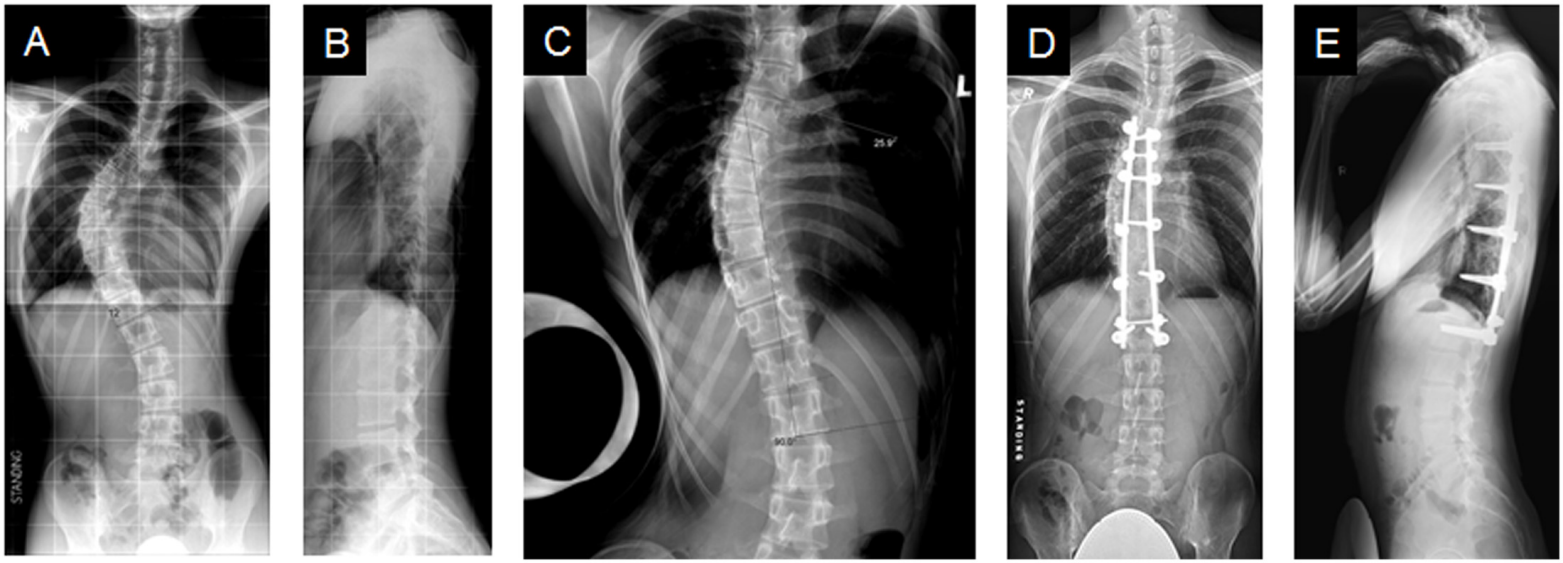
Saved distal fusion levels. A case where one level was saved. **(A)** A male AIS patient with a preoperative standing coronal Cobb angle of 61.6 degrees from T5-T12. **(B)** His standing sagittal Cobb angle from T5-T12 was 5.1 degrees. **(C)** Fulcrum bending radiograph demonstrated a curve of 31.3 degrees. Last follow-up **(D)** standing coronal Cobb angle was 26.8 degrees and **(E)** standing sagittal Cobb angle was 4.5 degrees.

**Fig 4:**
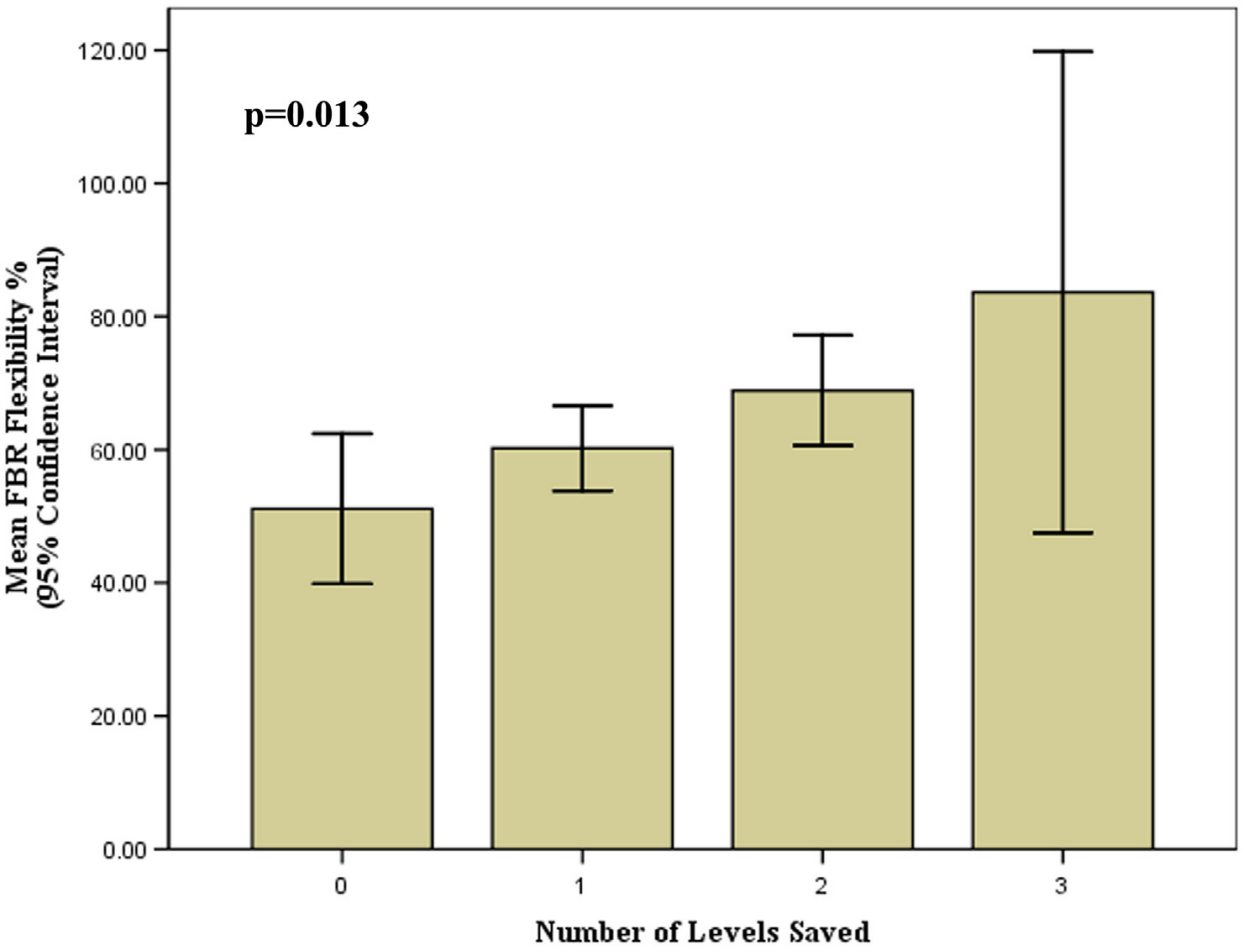
Bar graph illustrating the number of levels saved in relation to the mean fulcrum bending radiograph (FBR) flexibility percentage.

A total of 236 levels were fused. Eighteen patients (64.3%) out of 28 had distal levels saved in comparison to conventional methods (i.e. Harrington). A mean of 1.2 distal levels (range: 1–3 levels) were saved in 18 patients. Of all patients, 35.7% had no levels saved, 35.7% had one level saved, 21.4% had two levels saved, and 7.1% had three levels saved (Figs 2 and 3). Based on the number of levels saved, 0, 1, 2, and 3 levels corresponded to a mean FBR flexibility % of 51.1 (range: 26.8–78.7%), 60.2 (range: 47.2–76.9%), 68.9 (range: 56.0–78.0%), and 83.7 (range: 80.8–86.5%), respectively (p = 0.013) (Fig 4).

The mean immediate postoperative and last follow-up FBCIs were calculated as being 122.6 and 110.6%, respectively. From immediate postoperative to last follow-up at a 2 years, there was a statistically significant mean loss of 4.4 degrees (12% FBCI) from immediate postoperative to last follow-up coronal Cobb angle (p<0.001). However, the number of saved levels and curve flexibility were not factors found to be related to the loss of FBCI or curve correction on last follow-up (p>0.05). Sagittal alignment did not statistically differ between any assessment period and was maintained on last follow-up (p>0.05).

Trunkal shift was significantly reduced from a preoperative mean of 23.9mm to 15.3mm immediately postoperatively (p<0.01). At last follow up, the mean trunkal shift was 7.69 mm, which statistically differed compared to the immediate postoperative interval. The mean RSH was 7.7, 17.4, and 11.5mm at preoperative, immediate postoperative, and last follow-up intervals, respectively (p<0.01). The mean listing was 12.6, 13.6, and 10.7mm at preoperative, immediate postoperative, and last follow-up intervals, respectively. No statistical significant difference was noted between preoperative and last follow-up (p = 0.585), and between the immediate postoperative and last follow-up listing parameters (p = 0.733).

No patients were found to have evidence of add-on of other vertebra or curve decompensation necessitating revision to the next level down. There were no perioperative or postoperative instrumentation-related complications. On last follow-up, all radiographs demonstrated the presence of a fusion mass.

## Discussion

Spinal instrumentation is designed to correct spinal deformity, provide spinal stability, and achieve biological fusion. Before the advent of pedicle screws, Harrington [21] in the 1970s described a method of selecting fusion levels based on the concept of the "stable zone". This zone exists between two parallel lines drawn through the lumbosacral facets and the vertebral bodies positioned within these lines were classified as stable. During the same decade, King *et al* [20] suggested that more accuracy could be achieved by using the "central sacral line," which was defined as a line drawn through the centre of the sacrum perpendicular to the iliac crests. The level of fusion was determined as the vertebra that was bisected by this line, which was termed the "stable vertebra". Unfortunately, this meant that more levels were fused than not at the expense of spinal flexibility and motion preservation. For many years, these methods and others for selecting the fusion level were utilized; however, they were based on instruments that were available at the time of publication.

With the advent of pedicle screws and their inherent strength, it has been possible to preserve motion segments by fusing shorter while providing three dimensional correction. [7] Along with this change, some approaches at improving the assessment of spinal flexibility for selective thoracic fusion have been described (e.g. supine bending, traction and prone push radiographs). However, as previously mentioned, these radiographic techniques have some disadvantages in comparison to the FBR. The FBR utilizes static force, has no exposure to ionizing radiation to health-care worker, and allows preoperative consultation with the patient and family members with regards to the clinical-decision making process. Utilizing the FBR, we have reported the FBCI using hooks, hybrid systems, and with ALSS as 93%, 94%, and 122%, respectively.[23, 33] This high FBCI reflects the stronger power of using pedicle screws for curve correction. Understanding the postoperative predictive capacity of the FBR, we have further utilized it for selection of fusion levels. Based on our method of selecting fusion levels, we were able to obtain shorter fusions than the conventional Harrington method in 64.3% of our patients. Our study noted that a mean of 1.2 distal levels were saved. However, we were not able to obtain shorter fusions in 35.7% because their curves, based on the FBR, were more rigid.

Although the ALSS technique demonstrated a "statistically" significant coronal curve correction loss of a mean 2.8 degrees at two year follow-up, the coronal balance improved significantly from baseline. After further analysis, the number of saved levels and curve flexibility were not found to influence this loss of correction. Studies have reported that there is a 5% measurement error attributed to repeat Cobb angle measurements on x-rays.[39] As such, although we noted a "statistically" significant difference in curve correction from the immediate postoperative period to last follow-up, this loss was not "clinically" significant and well within the limits of measurement error. In addition, FBCI for ALSS is higher than hook or hybrid systems. We have demonstrated that by adopting this screw strategy and factoring in the predictive postoperative FBCI that also accounts for curve flexibility, the maximum number of motion segments is preserved (i.e. shorter fusions), trunk shift is minimized and with this the possibility of adjacent level degeneration may be reduced. [25] Furthermore, as our study noted, the more flexible the curve as determined by the FBR, the more levels can be saved (Fig 4) with no add-on of other vertebra or decompensation.

Although our study expands on the understanding of the concept of selection of fusion levels based on the FBR utilizing a pedicle screw construct, there are some limitations. For one, the sample size is not relatively large. Additional larger studies are needed to further validate our findings. Although no add-on phenomena was noted, long-term assessment of the patients, especially those that have not reached maturity, is ongoing. However, our study, which was retrospective with prospective data collection, noted similar FBCI measurements for ALSS as did other larger studies,[23] suggesting that the findings may demonstrate a true effect. Furthermore, longitudinal follow-up with advanced imaging utilizing this method of selection of fusion levels with ALSS is needed to assess the integrity of the adjacent segments and maintenance of curve correction. Nonetheless, our two-year follow-up of the largest series to date assessing patients in the context as described in our study provides insightful information, noting clinically-relevant maintenance of the curve, fusion, and no instrumentation-related complications.

## Conclusions

Based on one the largest series to date to assess curve flexibility via the FBR and the selection of fusion levels in AIS patients undergo pedicle screw fixation with a minimum of two-year follow-up, the results of this study suggest that the FBR can be used to determine fusion levels and its postoperative utility has been verified via ALSS in AIS patients. At two year follow-up, the FBR has facilitated the selection of the shortest fusion segment possible with 64.3% of the cases having at least one distal level saved with no add-on phenomena. Deformity correction and preservation of motion segments was achieved and clinically maintained, yielding successful fusion with no instrumentation or operative-related complications. Therefore, by assessing the flexibility of the curve with the FBR, this may dictate the degree of curve correction and provide an option for fusing shorter, in particular in AIS patients who present with flexible curves. In addition, our concept of selection of fusion levels in pedicle screw constructs can be utilized to compare outcomes between different fixation strategies for surgical treatment of AIS.
