# Correction: Selection of fusion levels using the fulcrum bending radiograph for the management of adolescent idiopathic scoliosis patients with alternate level pedicle screw strategy: clinical decision-making and outcomes

**DOI:** 10.1371/journal.pone.0138960

**Published:** 2015-09-18

**Authors:** The PLOS ONE Staff

The Corresponding Author in the author list is incorrect. The publisher apologizes for the error. The 8^th^ and 9^th^ authors, Keith D.K. Luk and Kenneth MC Cheung should be noted as corresponding authors. Dr. Luk may be contacted at hrmoldk@hku.hk. Dr. Cheung may be contacted at cheungmc@hku.hk.
